# The emerging role of WISP proteins in tumorigenesis and cancer therapy

**DOI:** 10.1186/s12967-019-1769-7

**Published:** 2019-01-16

**Authors:** Yi Liu, Yizuo Song, Miaomiao Ye, Xiaoli Hu, Z. Peter Wang, Xueqiong Zhu

**Affiliations:** 1 Department of Obstetrics and Gynecology, The Second Affiliated Hospital of Wenzhou Medical University, No. 109 Xueyuan Xi Road, Wenzhou, 325027 Zhejiang China; 2 Center of Scientific Research, The Second Affiliated Hospital of Wenzhou Medical University, Wenzhou, 325027 Zhejiang China; 3 Department of Biochemistry and Molecular Biology, School of Laboratory Medicine, Bengbu Medical College, Bengbu, 233030 Anhui China; 4 Department of Pathology, Beth Israel Deaconess Medical Center, Harvard Medical School, 330 Brookline Ave, Boston, MA 02215 USA

**Keywords:** Cancer, WISP, Oncogene, Drug, Targets, Therapy

## Abstract

Accumulated evidence has demonstrated that WNT1 inducible signaling pathway protein (WISP) genes, which belong to members of the CCN growth factor family, play a pivotal role in tumorigenesis and progression of a broad spectrum of human cancers. Mounting studies have identified that WISP proteins (WISP1-3) exert different biological functions in various human malignancies. Emerging evidence indicates that WISP proteins are critically involved in cell proliferation, apoptosis, invasion and metastasis in cancers. Because the understanding of a direct function of WISP proteins in cancer development and progression has begun to emerge, in this review article, we describe the physiological function of WISP proteins in a variety of human cancers. Moreover, we highlight the current understanding of how the WISP protein is involved in tumorigenesis and cancer progression. Furthermore, we discuss that targeting WISP proteins could be a promising strategy for the treatment of human cancers. Hence, the regulation of WISP proteins could improve treatments for cancer patients.

## Introduction

**Fig. 1:**
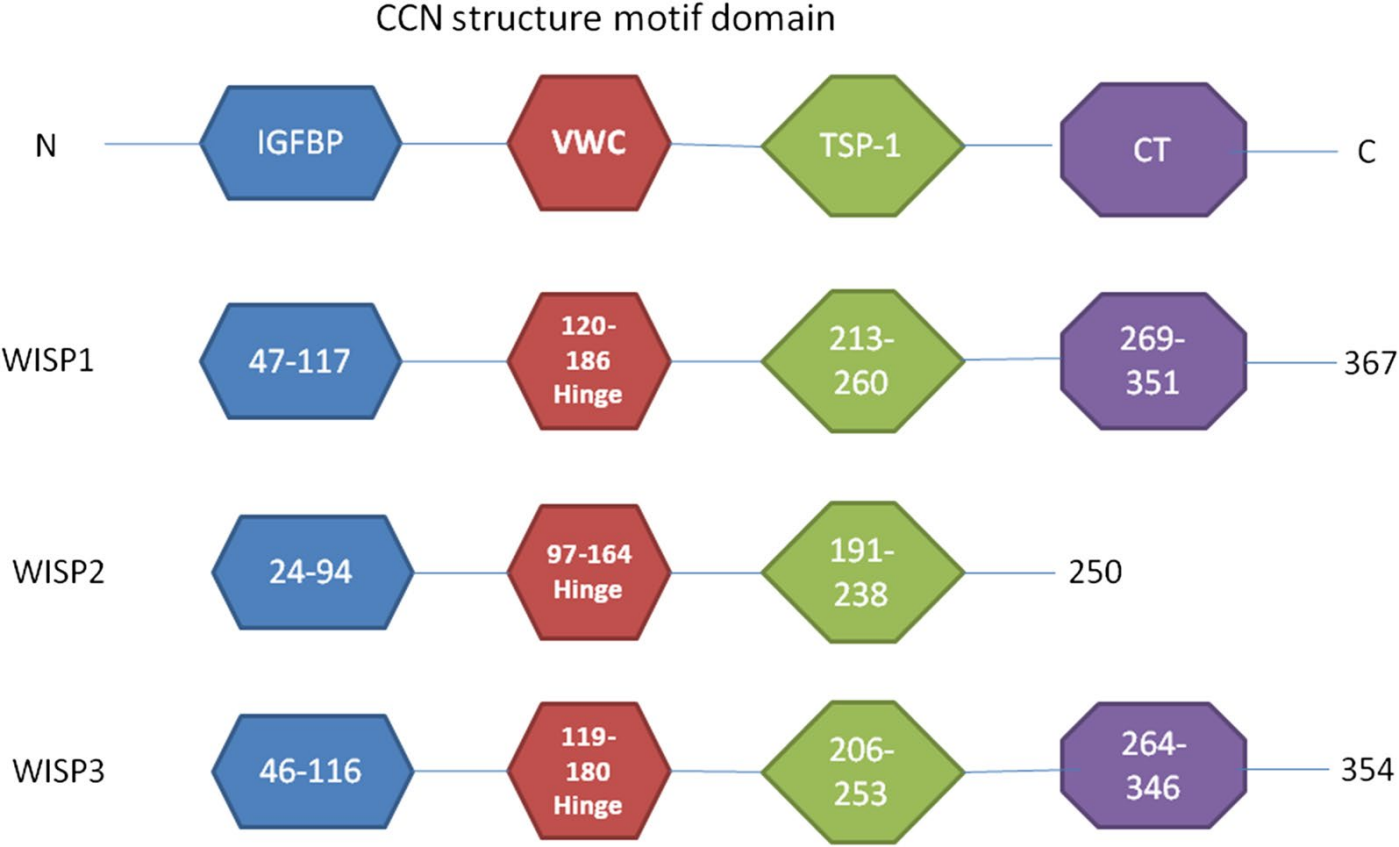
A schematic diagram of the structure of WISP proteins. WISP proteins have four highly conserved cysteine-rich motifs, which include the N-terminal motif, the von Willebrand factor-like (VWC) motif, the thrombospondin type 1 (TSP-1) motif, and the carboxy-terminal (CT) motif. The N-terminal motif consists of the first 12 cysteine residues and the IGF binding consensus sequence (GCGCCXXC). VWC and TSP1 motifs are involved in cell–cell interactions and angiogenesis inhibition. The CT motif forms a cysteine knot. WISP2 lacks the CT module

The connective tissue growth factor/cysteine-rich 61/nephroblastoma overexpressed (CNN) growth factor family includes cysteine-rich 61/CCN1 (CYR61), connective tissue growth factor/CCN2 (CTGF), nephroblastoma overexpressed/CCN3 (NOV), and WNT1 inducible signaling pathway protein (WISP) genes [1]. WISP genes were named because they are upregulated in mammary epithelial cells transformed by the Wnt-1 oncogene [2]. There are three identified WISP genes, which includeWISP1/CCN4, WISP2/CCN5, and WISP3/CCN6. CCN proteins have four highly conserved cysteine-rich motifs as follows: the N-terminal motif, the von Willebrand factor-like (VWC) motif, the thrombospondin type 1 (TSP-1) motif, and the carboxy-terminal (CT) motif [3] (Fig. 1). The N-terminal motif consists of the first 12 cysteine residues and the IGF binding consensus sequence (GCGCCXXC). VWC and TSP1 motifs could play a role in cell–cell interactions and angiogenesis inhibition. The CT motif forms a “cysteine knot”, which has been observed in other signaling peptides [3]. WISP2 lacks the CT module, which might lead to different functions than WISP1 and WISP3.

**Fig. 2:**
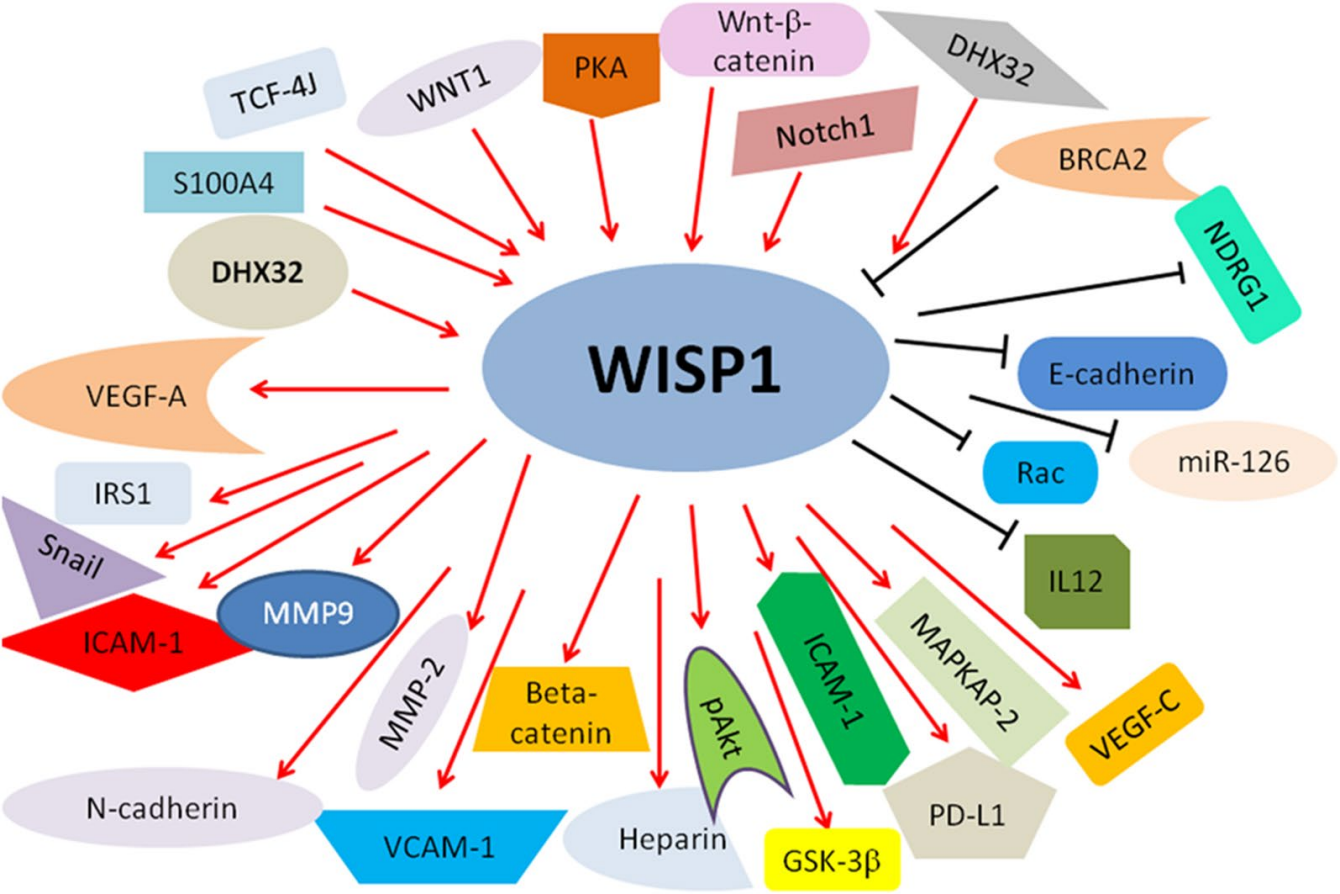
Illustrated pathways for WISP1-regulated downstream targets and the identified upstream regulators. “Arrow to WISP” → means “activating WISP”; “Arrows from WISP to → targets” means “activating targets”. “Blockade to WISP” means  “inhibiting WISP”; “Blockade from WISP to targets” meaning  “inhibiting targets”

**Fig. 3:**
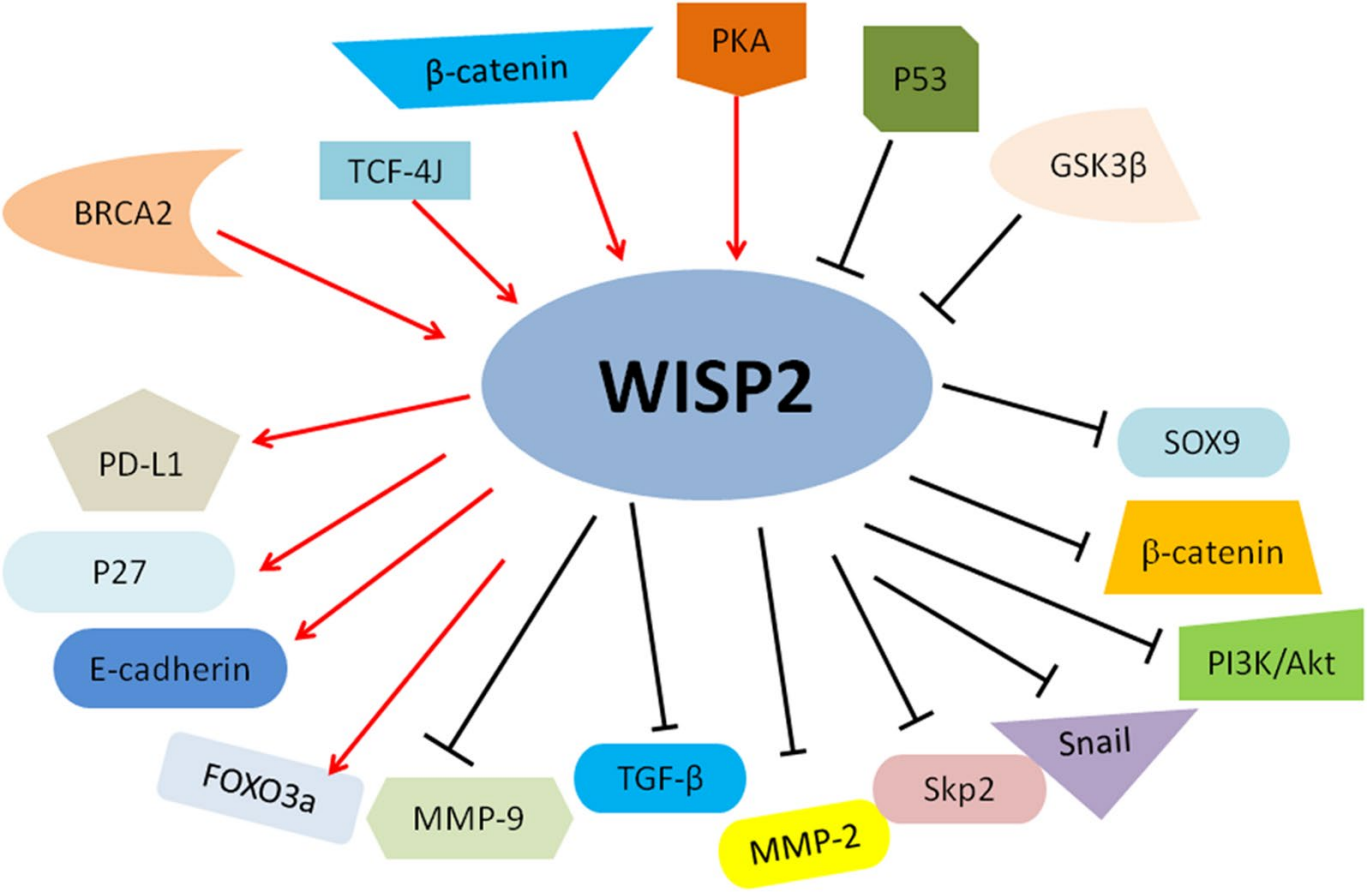
Illustrated pathways for WISP2-regulated downstream targets and the identified upstream regulators. “Arrow to WISP” → means “activating WISP”; “Arrows from WISP to → targets” means “activating targets”. “Blockade to WISP” means  “inhibiting WISP”; “Blockade from WISP to targets” meaning  “inhibiting targets”

**Fig. 4:**
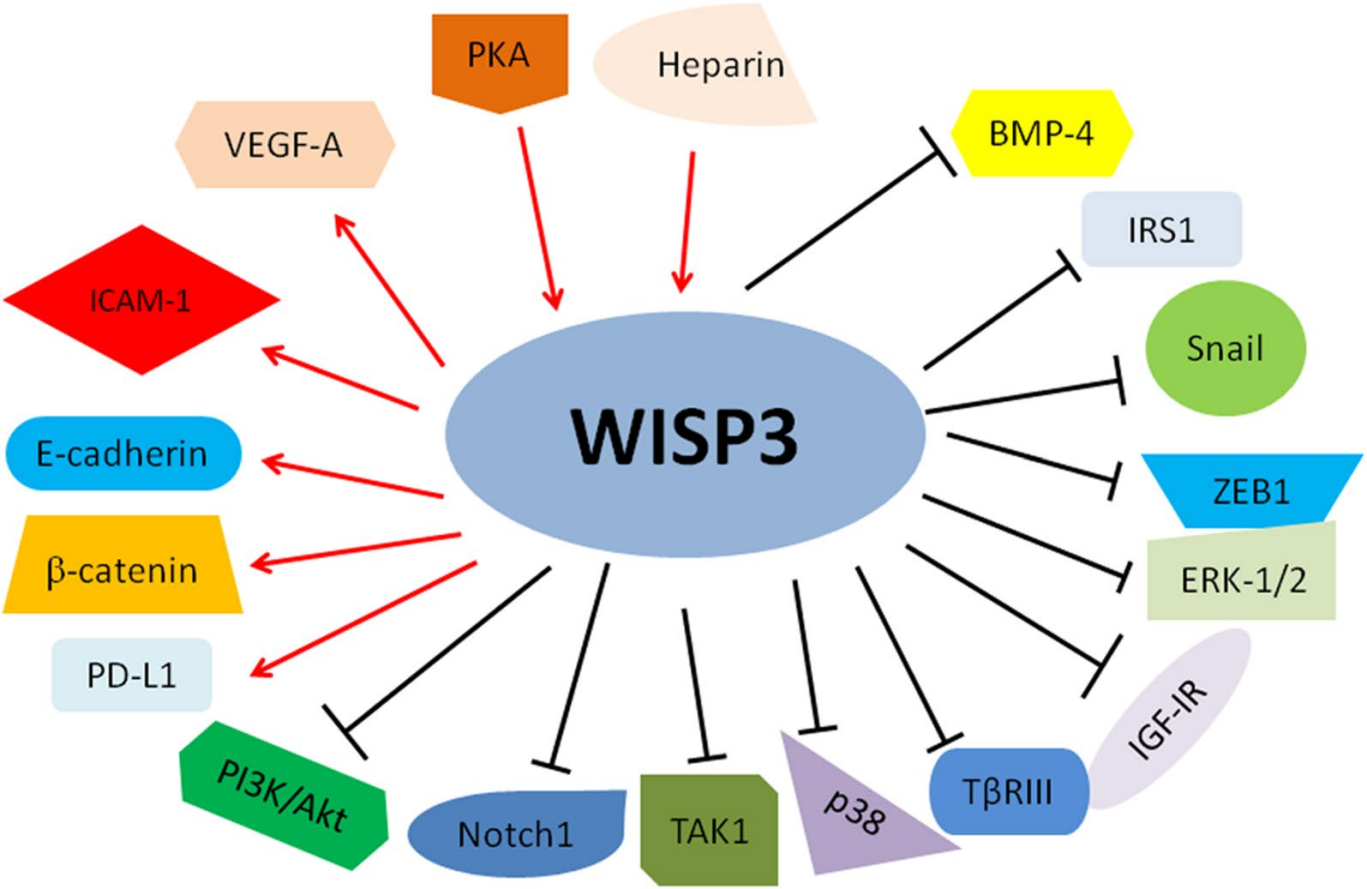
Illustrated pathways for WISP3-regulated downstream targets and the identified upstream regulators. “Arrow to WISP” → means “activating WISP”; “Arrows from WISP to → targets” means “activating targets”. “Blockade from WISP to  targets” meaning “inhibiting targets”

WISP1-3 genes are localized on the human chromosome 8q24.1–8q24.3, 20q12–20q13, and 6q22–6q23, respectively [2]. The role of WISP1-3 in human cancer is controversial. For instance, the RNA levels of WISP1 and WISP3 have been shown to be overexpressed in human colon tumors compared with normal mucoma, whereas WISP2 RNA expression has been shown to be reduced in colon tumors [2]. Another study reported that WISP1 mRNA expression was detected in normal and transformed breast cell lines. However, WISP2 mRNA was undetected in normal breast epithelial cells, but was observed in tumor-derived cell lines [4]. These reports suggest that WISP genes could have diverse functions in various human cancers. In the following sections, we highlight the physiological function of WISP proteins in a variety of human tumors. We further clarify the molecular mechanism underlying WISP-involved tumorigenesis and cancer progression. We also discuss whether targeting WISP could offer a promising strategy for the treatment of human cancers (Figs. 2, 3, 4).

## Role of WISP in human cancers

### Breast cancer

WISP1 has been reported to play an important role in breast cancer cells [4]. Xie et al. noticed the overexpression of WISP1 in primary breast tumors. Moreover, WISP1 expression is correlated with tumor stage, tumor size, and lymph node metastasis in breast cancer patients [5], suggesting that WISP1 exerts pro-tumorigenic functions in breast cancer. Consistent with this finding, Chiang et al. found similar results, in which WISP1 was highly expressed in breast cancer patients. Using an in vitro model, recombinant WISP1 treatment increased cell proliferation of breast cancer cells [6]. Overexpression of WISP1 enhanced cell growth and promoted tumor growth in mice. Strikingly, overexpression of WISP1 induced epithelial–mesenchymal transition (EMT) and changed the expression of EMT markers in breast cancer cells [6]. For example, WISP1 overexpression inhibited E-cadherin, but increased N-cadherin, Snail, and β-catenin. WISP1 upregulation also repressed the expression of the tumor suppressor NDRG1 in breast cancer cells [6]. In support of the oncogenic role of WISP1, one report demonstrated that increased WISP1 was associated with oncogenic transformation in invasive breast cancer. Importantly, WISP1 could be a paracrine inhibitor of type 1 cell-mediated immunity via suppression of IL-12 signaling and promotion of type 2 immunity to promote the transformation of invasive breast cancer [7]. Furthermore, WISP1 was increased after PTEN knockdown in MCF-7 breast cancer cells, leading to the promotion of cell migration and invasion [8]. Interestingly, another study showed that WISP-1 transcripts were observed in lower levels in breast cancer patients with poor prognosis [9], indicating that WISP1 seems to act as tumor suppressor in breast cancer. Consistent with this finding, the level of WISP1 expression was much lower in metastatic breast cancer compared to nonmetastatic breast cancer, suggesting that WISP1 may serve as an indicator of the risk of metastasis in breast carcinoma [10]. Therefore, an in-depth investigation is required to determine the biological and physiological functions of WISP1 in breast cancer.

WISP2 expression was markedly increased in human breast cancer cells after 17 β-estradiol (E2) treatment. This increase in WISP2 mRNA expression was completely inhibited by coincubation with an anti-estrogen agent, indicating that WISP2 could be directly regulated by estrogen receptors in human breast cancer cells [11]. This group further defined WISP2 as a secreted protein and as a marker of estrogen exposure in breast cancer cells [12]. WISP2 induction is highly specific for hormones that interact with the estrogen receptor [12]. Consistent with this report, the WISP2 gene exhibited estrogen- and progesterone-inducible expression and regulation of tumor cell proliferation in breast cancer cells [13]. WISP2 expression has been induced by protein kinase A (PKA) activators in human breast cancer cells [14]. Subsequently, one study confirmed that WISP2 expression can be modulated by serum and is correlated with serum-induced cell growth in breast cancer cells, demonstrating that WISP2 is responsible for serum stimulation and enhances cell growth in breast cancer [15]. WISP2 expression has been found to be highly overexpressed in human breast cancer MCF-7 cells, whereas it was undetectable or minimally detectable in nontransformed human mammary epithelial cells [15]. Along with this finding, WISP2 exhibits high levels of expression in breast cancer patients at late stages with metastasis, suggesting that WISP2 could be an oncoprotein in breast cancer [9].

One study showed that WISP2 expression is undetectable in normal and poorly differentiated breast cancer samples, but it is increased in noninvasive breast lesions [16]. The WISP2 expression level is inversely correlated with lymph node metastasis. In support of this correlation, WISP2 was found to be a negative regulator of migration and invasion via regulation of Snail, E-cadherin, matrix metalloproteinase-2 (MMP-2), and MMP-9 in breast cancer cells [16]. This study implied that WISP2 overexpression could have therapeutic implications for anti-invasion in breast cancer. Mechanistically, the overexpression of p53 mutants has been shown to inhibit WISP2 expression, enhance cell invasiveness, and induce EMT in ER-positive noninvasive breast tumor cells [17]. Notably, the depletion of WISP2 increased PD-L1 levels in breast cancer cells. The depletion of ZEB1 or overexpression of miR-200 family members in breast cancer cells with WISP2 depletion decreased PD-L1 expression levels. Inhibition of PD-L1 restored the susceptibility of resistant cells with WISP2 depletion to CTL-mediated killing apoptosis [18]. Additionally, the activation of WISP2 caused cell cycle arrest at the G1/G1 phase, reduced cell growth, and slowed tumor growth in a xenograft model via p27 upregulation and relocalization from the cytoplasm to the nucleus in breast cancer cells [19]. A mechanistic study showed that WISP2 suppresses Skp2 expression and subsequently stabilizes p27 accumulation, inducing FOXO3a expression and activity via shifting to the nucleus from the cytoplasm. Moreover, WISP2 inhibits the PI3K/Akt signaling pathway to dephosphorylate FOXO3a for its nuclear relocalization and activation [19]. These reports demonstrate that activation of WISP2 could have the potential for breast cancer treatment. Another study showed that knockdown of WISP2 promoted EMT in breast cancer cells, whereas overexpression of WISP2 reversed cells toward a more differentiated phenotype. Overexpression of WISP2 reduced cell proliferative and invasive phenotypes in breast cancer cells. This demonstrated that WISP2 could be involved in tumor cell invasion and metastasis in breast cancer [20]. This group further discovered that loss of WISP2 enhances the stem-like phenotype of cancer cells, which is characterized by high Cd44 expression, high aldehyde dehydrogenase (ALDH) activation and mammosphere development in breast cancer cells [21]. Consistently, WISP2 depletion increased the expression of the stem cell markers Nanog and Oct3/4, and activated the TGF-β pathway in breast cancer cells [21].

As early as 1999, WISP3 was identified to be underexpressed in inflammatory breast cancer (IBC) cell lines [22]. Subsequently, many studies further confirmed that WISP3 is downregulated in breast cancer cells and breast cancer tissues [23–25]. Consistent with these observations, inhibition of WISP3 expression promoted cell growth, invasion, and metastasis and protected cells from apoptosis and anoikis [25–28]. Kleer et al. revealed that WISP3 inhibits the expression of IGF-IR and its downstream targets, IRS1 and ERK-1/2, leading to cellular growth inhibition of breast cancer cells [29]. Similarly, inhibition of WISP3 expression induced EMT, enhanced cell growth, increased motility and invasiveness, and sensitized cells to IGF-1 treatment in human mammary epithelial cells [26]. Suppression of WISP3 has been shown to decrease the expression of E-cadherin via inducing Snail and ZEB1 in breast epithelial cells [25]. Consistent with this result, another research group identified that WISP3 inhibits ZEB1-triggered EMT and invasion through attenuation of IGF-1 receptor signaling in breast cancer [30]. Huang et al. found that low expression of WISP3 is correlated with high levels of phospho-Akt-1 in invasive breast carcinomas. WISP3 knockdown has been shown to activate the phosphatidylinositol 3-kinase (PI3K)/Akt pathway to enhance the survival of breast cancer cells [28]. Notably, WISP3 suppresses the invasion and metastasis of breast cancer cells through the inhibition of the bone morphogenetic proteins (BMP4) signaling pathway and subsequent suppression of TAK1 and p38 kinases [31]. Later, this group highlighted that knockdown of WISP3 can disrupt the acinar organization of breast cells via overexpression of type III TGF-β receptor (TβRIII), suggesting that WISP3 plays a role in maintaining acinar organization in breast tissue [32]. In a recently published study, WISP3 overexpression was sufficient to reverse EMT to MET and to reduce tumor initiating cells (TICs) via downregulating Notch1 signaling in breast cancer cells [33]. Strikingly, the results from a mouse model of mammary epithelial-specific WISP3 deletion indicated that the depletion of WISP3 in mice caused invasive high grade mammary carcinomas, which recapitulated human metaplastic breast carcinomas [34]. This mouse model provided direct evidence for the tumor suppressive role of WISP3 in breast cancer.

### Hepatocellular carcinoma (HCC)

Emerging evidence has revealed that the WISP family is critically involved in hepatocellular tumorigenesis. For example, WISP1 and WISP3 have been reported to be expressed in HCC cell lines. Moreover, two new variants generated by alternative splicing of WISP1 and WISP3 have been discovered, although the important function of these variants has not been elucidated in HCC [35]. Using c-Myc/E2F1 transgenic mouse model-driven liver cancer, the Wnt/β-catenin pathway, including its downstream targets, such as WISP1 and glutamine synthetase, has been activated, which was shown to confer cell growth in HCC [36]. One group provided direct evidence of WISP1 biological function in HCC cells. Inhibition of WISP1 suppressed cell proliferation, migration, and adhesion, but enhanced apoptosis via downregulation of pAkt, glycogen synthase kinase-3β (GSK-3β), and MMP-2 in HCC [37]. Interestingly, WISP1 was observed to be decreased in HCC samples and was closely associated with clinical features, such as invasion, pTNM stage, and patient survival, indicating that WISP1 may have a potential tumor suppressive function in HCC [38]. Undoubtedly, further studies are necessary to clarify the physiological function of WISP1 in HCC by in vitro and in vivo experiments.

Two studies reported that the hepatitis C virus core protein promotes cell growth via upregulation of Wnt-1 expression and its downstream gene WISP2 in HCC cells, indicating that WISP2 could play a potential role in HCC cell growth [39, 40]. HBx mutants, which frequently occur in hepatitis B virus (HBV)-related hepatocellular carcinoma, have been reported to promote cell proliferation and migration through the activation of the Wnt/β-catenin signaling pathway [41]. Specifically, HBx mutants increased and stabilized β-catenin levels via inhibition of GSK3β in HCC cells, leading to the upregulation of WISP2 and c-Myc [41]. Similarly, the T-cell factor 4 (TCF-4) transcription factor protein has been shown to regulate the Wnt/β-catenin signaling pathway in human cancers [42, 43]. The expression level of WISP2 is enhanced in HCC cells overexpressing TCF-4J (TCF-4 isoform J), suggesting that WISP2 could be involved in HCC progression [44]. One group found that WISP2 expression does not differ between HCC samples and normal liver tissues, while WISP3 was only detected in a few HCC samples [37]. Therefore, it is necessary to clarify the role of WISP2 and WISP3 in HCC in the near future.

### Colon cancer

Increasing evidence has supported the important roles of the WISP family in colon tumorigenesis. For example, WISP1 has been found to be upregulated in sporadic colon carcinomas, indicating that WISP1 might be an oncoprotein in colon cancer [2, 45]. In vitro experiments have shown that indomethacin induces apoptosis and inhibited proliferation partly via downregulation of WISP1 in colon cancer cells [46]. In addition, overexpression of DHX32 increased the expression of WISP1, MMP-7 and VEGFA through activation of the Wnt pathway, resulting in colon cancer progression and metastasis [47]. Downregulation of WISP1 caused inhibition of cell growth and invasion, induction of apoptosis and cell cycle arrest at G1 phase in colon cancer cell lines [48]. In vivo experiments have shown that inhibition of WISP1 slows the progress of tumor formation. WISP1 expression has been shown to be substantially increased in colon cancer tissues and is associated with advanced pathological stage and poor prognosis [48]. This integrated analysis supports that WISP1 could be an oncogene in colon cancer. An aberrant level of WISP2 expression has been observed in colon cancer. Interestingly, WISP2 DNA expression has been shown to be amplified, but RNA expression is reduced in most colon tumors [2]. One group reported that WISP-3 is overexpressed in human colon tumors [2]. However, another group found that WISP3 harbors loss of expression, frameshifts, and mutations in colon cancer, suggesting that WISP3 could play a tumor suppressive role in colon cancer [49]. Due to limited studies on WISP2 and WISP3, it is necessary to determine the role of WISP2 and WISP3 in colon cancer development and progression.

### Gastric cancer

The WISP family has been determined to be involved in gastric tumorigenesis. WISP1 expression levels have been shown to be upregulated in gastric cancer tissues. Inhibition of WISP1 leads to decreases in cell proliferation, migration and invasion in gastric cancer cells via suppression of cyclin D1 and EMT progression, demonstrating that WISP1 could be an oncogene in gastric cancer [50]. Emerging evidence has shown that WISP2 plays a critical role in the development of gastric cancer. Consistent with this role, the expression of WISP2 is associated with tumor stage, differentiation status, and overall survival [51]. The depletion of WISP2 has been shown to enhance cell growth, migration and invasion, whereas overexpression of WISP2 inhibits cell metastasis via regulating EMT and downregulating the expression and activity of MMP-9 and MMP-2 through JNK and ERK in gastric cancer cells [51]. This study suggested that WISP2 could be a potential tumor suppressor in gastric cancer. However, another study validated that WISP2 is highly expressed in gastric tissues compared to adjacent normal tissues [52]. It has been reported that WISP2 could regulate the Wnt/β-catenin signaling pathway in gastric cancer cells. Moreover, WISP2 and β-catenin exhibit a positive correlation in gastric cancer patients, which was observed in patients with gastric cancer at early stages [52]. Thus, the expression of WISP2 and β-catenin could be a biomarker for the prediction and prognosis of gastric cancer patients.

WISP3 has been identified to exhibit mutations and loss of expression in gastric cancer, indicating the antitumor role of WISP3 in gastric cancer [49]. Consistently, the WISP3 gene is mutated by approximately 11–20% in microsatellite unstable gastric carcinoma [53]. Interestingly, high WISP3 high expression levels are closely associated with gastric cancer size and tumor invasion, indicating that WISP3 could be an oncoprotein in gastric cancer [54]. Consistent with this characteristic, the depletion of WISP3 has been shown to inhibit cell proliferation, migration and invasion via prevention of β-catenin transfer from the cytoplasm to the nucleus and subsequent suppression of Wnt/β-catenin signaling and its downstream genes in gastric cancer [54]. This finding suggests that WISP3 could be a potential therapeutic target for gastric cancer.

### Pancreatic cancer

Several groups have explored the role of the WISP family in the progression of pancreatic cancer. For instance, the down-regulation of BRCA2 has been shown to promote malignant transformation partly via increased WISP1 and decreased WISP2 expression in pancreatic cancer cells [55]. This study only indicates the potential role of WISP1 in pancreatic cancer. Interestingly, there is no direct evidence to show the physiological function of WISP1 in pancreatic cancer. WISP2 expression has been detected in normal and cancerous epithelial cells of the pancreas [56]. WISP2 mRNA expression was undetected in pancreatic cancer stromal cells [56]. Previous studies have suggested that WISP2 has a pathological role in the regulation of pancreatic cancers via regulating microenvironments. WISP2 expression is downregulated in pancreatic cancer tissues [57]. The WISP2 recombinant protein promotes mesenchymal–epithelial transition (MET) in pancreatic cancer cells. Moreover, loss of WISP2 expression has been observed in p53 mutant pancreatic cancer cell lines, indicating that p53 could regulate the expression of WISP2 in pancreatic cancer cells [57]. Notably, Wu et al. reported that protein phosphate 2A (PP2A) inhibitors inhibit cell growth and migration through suppression of the Wnt/β-catenin pathway, including WISP2, in pancreatic cancer cells [58]. Because the function of WISP1 and WISP3 has not been determined in pancreatic cancer, it is recommended to investigate the biological function and molecular mechanism of the WISP family in pancreatic carcinogenesis and progression.

### Lung cancer

The WISP family has been involved in the progression of lung cancer. It has been shown that WISP1 overexpression decreases cell motility and invasion via suppression of Rac activity in lung cancer cells [59]. Controversially, several reports have implied an oncogenic role of WISP1 in lung cancer. Matsubara et al. found that S100A4 upregulates WISP1 expression, which could be involved in lung cancer progression [60]. Similarly, WISP-1 was found to be upregulated in primary lung cancer tissues, which is correlated with tumor histology [61]. Interestingly, WISP1 has been reported to have a total of twenty-eight polymorphisms, which are correlated with the chemotherapeutic toxicity, hematologic toxicity and gastrointestinal toxicity in lung cancer. These genotypes of WISP1 may provide a potential for further clinical application of personalized diagnosis and platinum-based chemotherapy in lung cancer patients [62, 63]. Additionally, overexpression of WISP1 has been shown to contribute to pulmonary metastases in D122 Lewis lung cancer mouse models [64]. WISP2 exhibits higher methylation levels in cisplatin (DDP)-resistant lung cancer tumors, which tend to be associated with adverse overall survival, implying that WISP2 may play a potential role in cisplatin resistance [65]. Furthermore, epigallocatechin-3-gallate (EGCG), which is the primary polyphenol in green tea, has been shown to reverse DDP resistance via demethylation of WISP2 in lung cancer cells [66]. Only one study identified the WISP3 mutation in early non-smoking lung cancer patients [67], indicating the involvement of WISP3 in lung cancer. In-depth investigations are required to address the detailed functions of the WISP family in lung cancer development and progression.

### Melanoma

WISP1 has been reported to promote pulmonary metastases in melanoma cell lines, suggesting that WISP1 plays a pivotal oncogenic role in melanoma [64]. However, decreased expression of WISP1 has been detected in melanoma nests compared to the adjacent normal dermis [68]. Overexpression of Notch1 signaling in stromal fibroblasts has been shown to suppress cell growth via upregulation of WISP1 in melanoma [68]. Moreover, WISP1 has been shown to be aberrantly expressed in both melanoma cells and the tumor periphery by inhibiting their response to IL12 to exert paracrine action on immune cells [69]. Furthermore, another study demonstrated that the Notch1-WISP-1 axis governs stromal regulation of melanoma metastasis, indicating that this axis could be a potential target for the treatment of melanoma metastasis [70]. By fluorescence in situ hybridization technology, melanoma samples have shown decreases in copy numbers near or at WISP3 [71]. To date, studies regarding the role of WISP2 and WISP3 are not available; therefore, the function of WISP must be determined in the near future.

### Prostate cancer

Prostate cancer is a leading cause of death in males. The WISP family is linked to prostate cancer progression. The level of WISP1 expression is upregulated in prostate cancer tissues, especially at early stages. Anti-WISP1 antibody treatment has led to the inhibition of prostate cancer cell growth [72]. Consistent with this result, osteoblast-derived WISP1 has been shown to promote migration and expression of VCAM-1 by downregulating miR-126 via αvβ1 integrin, FAK, and p38 signaling pathways [73]. Recently, it has been reported that osteoblast-secreted WISP-1 enhances cell adhesion to bone through the VCAM-1/α4β1 integrin pathway in prostate cancer cells [74].

### Oral carcinoma

The WISP family has been identified to be associated with oral tumorigenesis. An in vitro experimental study has shown that knockdown of WISP1 induces apoptosis and inhibits the invasion in oral squamous cell carcinoma (OSCC) cells [75]. Increased WISP1 expression has been detected in tissues of OSCC patients and is correlated with poor survival and treatment failure in human OSCC [75]. WISP1 promotes cell migration and increases intercellular adhesion molecule-1 (ICAM-1) expression through the αvβ3 integrin receptor and the ASK1, JNK/p38, and activator protein 1 (AP-1) pathways in OSCC cells [76]. Clinically, WISP-1 expression has been associated with the tumor stage in OSCC patients [76]. This group further determined that WISP1 enhances angiogenesis by promoting VEGF-A expression. Consistently, WISP1 expression is associated with VEGF-A levels in OSCC specimens. Mechanistically, WISP-1 upregulates VEGF-A expression through the αvβ3 integrin/FAK/c-Src pathway, and subsequently activates the EGFR/ERK/HIF-1α pathway in OSCC [77]. Moreover, high expression of WISP1 due to its hypomethylation has been observed in OSCC patients with lymph node metastasis. High WISP1 expression is correlated with lymph node (LN) metastasis and survival, implying that WISP1 could be a potential biomarker for predicting LN metastasis in OSCC [78]. Consistent with this observation, WISP1 increases the expression of VEGF-C via inhibition of miR-300 and regulation of the αvβ3 integrin/integrin-linked kinase/Akt pathway in OSCC. Indeed, the correlation between WISP1 and VEGF-C in OSCC tissues has been identified [79]. Another study demonstrated that the WISP1 rs2929970 polymorphism is susceptible to OSCC in individuals who smoke, whereas WISP1 rs16893344 is causally linked to the occurrence of OSCC in betel nut chewing individuals [80]. This finding indicates that the WISP1 polymorphism could be a marker or treatment target in OSCC. It has been illustrated that the amplification of WISP1 is associated with a significantly decreased survival in oropharyngeal squamous cell carcinomas (OPSCC) [81]. One report has revealed a higher level of WISP2 expression in salivary gland tumor-derived cell lines [82]. There are no available studies referring to the function of WISP3 in oral carcinoma. Therefore, it is warranted to clarify the role of WISP2 and WISP3 in oral carcinoma in the near future.

### Esophageal squamous cell carcinoma (ESCC)

WISP-1 overexpression has been shown to enhance cell growth in ESCC cell lines and is closely correlated with tumor size, tumor type, lymph node metastasis and poor prognosis of ESCC [83], suggesting that WISP1 could be a clinical biomarker for a poor prognosis in ESCC. Similarly, another group validated that WISP1 is highly expressed and associated with overall survival in ESCC patients [84]. WISP-1 overexpression has also been shown to be associated with radioresistance in esophageal cancer cells and mice during fractionated irradiation, while depletion of WISP1 reverses radioresistance and leads to cell death [85]. Zhang et al. observed that WISP1 is involved in radioresistance mainly via inhibiting irradiation-mediated DNA damage and activation of PI3K kinase. Radiation treatment leads to the upregulation of BOKAS lncRNA and subsequently increases WISP1 expression, resulting in radioresistance. This study provided insight regarding WISP1 regulation in response to radiation, showing a positive feedback loop that causes radioresistance [84]. Notably, WISP1 has been shown to be highly expressed in radioresistant cells with an EMT phenotype in ESCC, suggesting that inhibition of WISP1 could be an attractive approach to overcome EMT-associated radioresistance in ESCC [86]. These reports clearly suggest that WISP1 plays an oncogenic role in ESCC and could be a potential target for treating ESCC. To date, the role of WISP2 and WISP3 in ESCC remains unelucidated.

### Osteosarcoma and chondrosarcoma

WISP1 has been identified as an osteogenic potentiating factor that enhances mesenchymal cell proliferation and induces osteoblastic differentiation, but suppresses chondrocytic differentiation, implying that WISP1 is a key molecule for bone development and fracture repair [87]. In support of these characteristics, WISP1 has a positive influence on bone cell differentiation and formation via enhancing BMP-2 activity [88, 89]. Due to the role of WISP1 in bone cell differentiation, dysregulated WISP1 could be involved in human osteosarcoma. In fact, two studies confirmed this role because WISP1 has been found to be highly expressed in osteosarcoma patients and is determined to be associated with tumor stage [90]. WISP1 promotes the migration of osteosarcoma cells through upregulation of MMP-2 and MMP-9 via several signaling pathways [90]. Recently, WISP-1 has been shown to increase VEGF-A expression and angiogenesis via regulation of the FAK/JNK/HIF-1α pathway, and inhibition of miR-381 expression in osteosarcoma [91]. It is necessary to explore the role of WISP2 and WISP3 in osteosarcoma tumorigenesis because there are no available reports that define the function of WISP2/3 in osteosarcoma.

WISP1 enhances cell migration by increasing MMP-2 expression by regulating the α5β1 integrin receptor, FAK, MEK, ERK, p65 and NF-κB signal transduction pathway in chondrosarcoma, suggesting that WISP1 may play an oncogenic role in chondrosarcoma [92]. Interestingly, two WISP1 variants (WISP1v and WISP1vx) have been identified in a human chondrosarcoma-derived chondrocytic cell line. Overexpression of WISP1v has been shown to enhance chondrocyte differentiation toward endochondral ossifications, while WISP1vx is involved in the transformed phenotypes of chondrosarcomas [93]. However, one study has shown that WISP1 expression is decreased in higher-grade chondrosarcomas [94], indicating that WISP1 function in chondrosarcoma is controversial and needs to be clarified. WISP3 promotes the migration of chondrosarcoma cells via upregulation of ICAM-1 expression by regulating multiple molecules, including αvβ3 and αvβ5 integrin receptors, FAK, MEK, ERK, c-Jun, and AP-1 [95]. In addition, WISP3 enhances VEGF-A production and induces angiogenesis by inhibiting miR-452, suggesting that WISP3 may be involved in cell differentiation and migration in chondrosarcoma [96].

### WISP in gynecological cancers and other tumors

Increased expression of WISP-1 has been observed in endometrial endometrioid adenocarcinoma (EEC) compared to that in the secretory endometrium. Strikingly, high expression of WISP-1 is associated with poor survival in EEC [97]. A significant correlation between genetic polymorphisms of WISP1 and invasive cervical cancer has been observed. Genotype AA in WISP1 SNP rs2977537 enhances the risk of invasive cervical cancer, whereas genotypes AG/GG in rs2977530 decrease the susceptibility to invasive cervical cancer [98]. Zheng et al. used cDNA and tissue microarrays to analyze the gene expression profiles in epithelial ovarian tumors and found that WISP2 is a new candidate oncogene for epithelial ovarian cancer [99].

WISP2 is downregulated in leiomyoma tissues compared to the normal myometrial counterparts [100]. Moreover, overexpression of WISP2 inhibits cell proliferation and motility in human myometrial and leiomyoma smooth muscle cells [100]. Interestingly, WISP2 is induced by heparin in vascular smooth muscle cells, but not in uterine smooth muscle cells [100]. This data indicated that loss of WISP2 expression in normal myometrium could contribute to leiomyomas. One study showed that WISP2 is overexpressed in the gastrointestinal peptide (GIP)-dependent corticotropin/ACTH-independent macronodular adrenal hyperplasia (AIMAH), suggesting that WISP2 could play a role in adrenocortical hyperplasias and tumors [101]. Inhibition of WISP2 has been observed in clear-cell renal cell carcinoma (ccRCC), indicating that WISP2 could be a tumor suppressor in ccRCC.

## Conclusion and perspective

In summary, WISP proteins are critically involved in various cellular progresses, including cell proliferation, apoptosis, invasion and metastasis in cancer cells. Moreover, WISP proteins play important roles through the regulation of their downstream targets in the development and progression of human cancers (Tables 1, 2, 3). Notably, WISP proteins could exhibit oncogenic or tumor suppressive functions in different tumor types. In this regard, targeting WISP proteins could be a novel approach for the treatment of human malignancies. It is necessary to design personalized medicine to target WISP proteins in specific tissues. Specifically, WISP inhibitors need to be developed for cancer patients with increased expression of the WISP oncoprotein. One alternative strategy is to regulate upstream effectors that control the expression of WISP proteins. We postulate that targeting WISP proteins for cancer therapy is intriguing.

**Table 1:** Role of WISP1 in human cancers

Cancer type	Function	Target	Reference
Breast cancer	Increases cell proliferation, migration and invasion; promotes tumor growth; induces EMT; promotes type 2 cell-mediated immunity; inhibits type 1 cell mediated immunity; suppresses tumor metastasis	Inhibits E-cadherin, NDRG1; increases N-cadherin, Snail, and β-catenin	[4, 6–10, 102]
Hepatocellular carcinoma (HCC)	Promotes cell growth; Inhibits WISP1; suppresses cell proliferation, migration, and adhesion; associated with invasion, pTNM stage, and patient survival	Induced by Wnt/β-catenin pathway; activates pAkt, GSK-3β, and MMP-2	[35–38]
Colon cancer	Promotes tumorigenesis, progression and metastasis; associated with advanced pathological stage and poor prognosis; harbors loss of expression, frameshifts and mutations	Induced by DHX32, Wnt pathway	[2, 45–48]
Gastric cancer	Promotes cell proliferation, migration and invasion	Activates cyclin D1	[50]
Pancreatic cancer	Promotes malignant transformation	Inhibited by BRCA2	[55]
Lung cancer	Retards cell motility and invasion; promotes cancer progression; correlated to tumor histology; correlated to chemotherapeutic toxicity; leads to pulmonary metastases	Increased by S100A4	[59–64]
Melanoma	Promotes pulmonary metastases; suppresses cell growth	Increased by Notch1	[64, 68–70]
Prostate cancer	Promotes cancer progression, migration, growth; associated with cancer stage; enhances cell adherence to bone	Increases VCAM-1; inhibits miR-126	[72–74]
Oral carcinoma	Promotes cell migration; correlated with the tumor stage and poor survival; enhances angiogenesis	Enhances ICAM-1, VEGF-A and VEGF-C; activates integrin αvβ3/FAK/c-Src and EGFR/ERK/HIF-1α pathways; inhibits miR-300	[75–81]
Esophageal squamous cell carcinoma	Enhances cell growth; associated with tumor size, tumor type, lymph node metastasis and poor prognosis; contributes to radioresistance	Not detecte	[83–86]
Osteosarcoma	Enhances bone cell differentiation and formation; associated with tumor stage; promotes cancer cell migration and tube formation	Enhances BMP-2, MMP-2 and MMP-9; improves VEGF-A; inhibits miR-381	[87–91]
Chondrosarcoma	Promotes cell migration	Upregulates MMP-2	[92–94]
Endometrial endometrioid adenocarcinoma	Associated with poor survival and clinical grades	Not detected	[97]

**Table 2:** Role of WISP2 in human cancers

Cancer type	Function	Target	Reference
Breast cancer	Exhibits estrogen and progesterone inducible expression; enhances tumor cell proliferation and growth; associated with metastasis and late stage; inhibits tumor growth, migration and invasion; causes cell cycle arrest at the G1/G1 phase	Induced by PKA; inhibits Snail, E-cadherin, MMP-2, MMP-9, Skp2, PI3K/Akt, TGF-β pathways; inhibited by p53 mutants; induces PD-L1, p27, and FOXO3a	[9, 11–15, 18–21, 103, 104]
Hepatocellular carcinoma	Promotes cell growth, proliferation and migration; involved in HCC progression	Increased by β-catenin, and TCF-4J; inhibited by GSK3β	[39–44]
Colon cancer	WISP2 DNA expression is amplified, but function remains unknown	Not detected	[2]
Gastric cancer	Associated with tumor stage, differentiation status, and survival; inhibits cell growth, migration and invasion	Downregulates MMP-9 and MMP-2 through JNK and ERK pathways	[51, 52]
Pancreatic cancer	Promotes mesenchymal–epithelial transition; regulates pancreatic cancers via regulating microenvironments	Promoted by BRCA2	[55–58]
Lung cancer	Associated with adverse overall survival	N/A	[65, 66]
Leiomyoma	inhibits cell proliferation and motility	N/A	[100]

**Table 3:** Role of WISP3 in human cancers

Cancer type	Function	Target	Reference
Breast cancer	Suppresses cell growth, invasion, metastasis; maintains acinar organization; reverses EMT to MET, reduces TICs (tumor initiating cells)	Inhibits IGF-IR, IRS1 and ERK-1/2, snail and ZEB1, BMP4, TAK1 and p38 kinase, PI3K/Akt, TβRIII, and Notch1; increases E-cadherin	[22–34]
Hepatocellular carcinoma (HCC)	Detected in only a few HCC samples, and its function remains unknown	Not detected	[37]
Colon cancer	Harbors loss of expression, frameshifts, and mutations	Not detected	[2, 49]
Gastric cancer	Exhibits mutations and loss of expression; associated with cancer size and tumor invasion; promotes cell proliferation, migration and invasion	Induces Wnt/β-catenin signaling	[49, 53, 54]
Lung cancer	Exhibits mutations in early nonsmoking lung cancer patients	Not detected	[67]
Melanoma	Shows loss of copy numbers		[71]
Chondrosarcoma	Promotes cell migration and differentiation	Upregulates ICAM-1and VEGF-A; inhibits miR-452	[95, 96]

The functions of WISP proteins have been revealed; however, several critical questions need to be addressed. For example, what are the WISP binding partners? Which receptors and signaling pathways are regulated by WISP? What are the direct targets of WISP? How does WISP govern their downstream targets? To address these concerns, using tissue-specific knockout mouse models or transgenic mice are advantageous approaches that could be used to understand the contribution of WISP proteins in carcinogenesis. In addition, the detection of pathological gene alterations of WISP in cancer patients is required. High-throughput sequencing technology could be helpful in determining the molecular mechanism of WISP-involved tumorigenesis. These in-depth studies could provide experimental evidence and a rationale for developing inhibitors of WISP proteins for cancer treatments.

## Abbreviations

WISP: WNT1 inducible signaling pathway protein
CNN: connective tissue growth factor/cysteine-rich 61/nephroblastoma overexpressed
CYR61: cysteine-rich 61/CCN1
CTGF: connective tissue growth factor/CCN2
NOV: nephroblastoma overexpressed/CCN3
VWC: von Willebrand factor-like motif
TSP-1: thrombospondin type 1
CT: carboxy-terminal
EMT: epithelial–mesenchymal transition
PKA: protein kinase A
MMP: matrix metalloproteinase
ALDH: aldehyde dehydrogenase
IBC: inflammatory breast cancer
PI3K: phosphatidylinositol 3-kinase
BMP4: bone morphogenetic proteins
TβRIII: type III TGF-β receptor
TICs: tumor initiating cells
HCC: hepatocellular carcinoma
GSK-3β: glycogen synthase kinase-3β
HBV: hepatitis B virus
TCF-4: T cell factor 4
MET: Mesenchymal–epithelial transition
PP2A: protein phosphate 2A
EGCG: epigallocatechin-3-gallate
OSCC: oral squamous cell carcinoma
ICAM-1: intercellular adhesion molecular-1
AP-1: activator protein 1
OPSCC: oropharyngeal squamous cell carcinomas
ESCC: esophageal squamous cell carcinoma
EEC: endometrial endometrioid adenocarcinoma
GIP: gastrointestinal peptide
AIMAH: corticotropin/ACTH-independent macronodular adrenal hyperplasis
ccRCC: clear-cell renal cell carcinoma
